# Structural Properties, Mechanical Behavior, and Food Protecting Ability of Chickpea Protein-Derived Biopolymer Films

**DOI:** 10.3390/polym17141938

**Published:** 2025-07-15

**Authors:** Mehmet Şükrü Karakuş

**Affiliations:** Application and Research Center for Science and Technology, Harran University, Sanliurfa 63300, Turkey; sukrukarakus@harran.edu.tr

**Keywords:** chickpea protein, functionalized edible film, FTIR spectra, mechanical behavior, food packaging

## Abstract

This study aimed to enhance the characteristic properties of chickpea proteins enriched with quercetin by incorporating whey proteins. For this, whey proteins were supplemented into the film systems at 10, 20, 30, 40, and 50% of the total protein content, and these formulations were labeled as CWF1, CWF2, CWF3, CWF4, and CWF5, in that order. Negative control (CF) was designed with chickpea protein alone. Essential amino acid content of chickpea protein (16.48%) was higher than that of whey protein (8.09%). FTIR spectra revealed protein–protein interactions occurred within film systems. Raising the whey protein content above 40% led to morphological issues in the films. Differences in moisture content, thickness, color, and opacity were obvious (*p* < 0.05). As the protein content boasted, a decrease in solubility and an increase in the swelling ratio of the films was detected (*p* < 0.05). CWF4 exhibited enhanced barriers and mechanical properties, followed by CWF3, CWF2, CWF1, CF, and CWF5 (*p* < 0.05). Moreover, in food simulators, quercetin release from films was monitored, and the highest release of quercetin occurred in 50% ethanol, followed by water and 95% ethanol. Ultimately, highly functional quercetin-loaded edible films, especially CWF4, stood out in protecting fresh strawberries.

## 1. Introduction

Synthetic plastic materials account for approximately 37% of the food packaging market thanks to their cost-effectiveness, portability, adaptability, strong mechanical strength, and high barrier properties [1,2]. However, prolonged contact between food and plastic packaging can cause microplastic particles to migrate into the food, potentially leading to their ingestion when the food is consumed [3]. Furthermore, merely 20% of plastic packaging materials are effectively recycled, whereas the remainder results in waste that adversely affects the environment [4].

In food packaging, edible films and coatings play a crucial role in reducing waste and offering strong resistance to moisture, aroma loss, solvent transfer, and respiration [5]. Biodegradable, non-toxic polysaccharides, lipids, and proteins are employed in the development of edible films or coatings [6,7]. Among these, protein-based films are particularly preferred due to their excellent processability, versatility, high nutritional value, and improved controllability [8]. Moreover, the diverse amino acid composition and complex conformational structures of proteins contribute significantly to the enhanced mechanical properties of such films [9]. Namely, proteins contain 20 amino acid monomers in their structures and participate in chemical reactions thanks to the bonds they have (covalent, disulfide, and peptide bonds), which makes them stand out in edible films [10]. In addition to these functional advantages, protein films also release nitrogen upon degradation, allowing them to act as fertilizers—an environmentally beneficial feature not typically observed in non-protein-based films [11]. Moreover, plant proteins utilized as raw materials in food packaging provide benefits in terms of their compatibility with vegan diets, active ingredients, variety, and economic sustainability [12]. This subject, the film-coating properties of soy protein [13], wheat gluten [6], and corn zein proteins [7], has been examined in various studies. While protein films provide advantages, certain challenges remain. Such films are known for their hydrophilic properties [14]. These characteristic properties indicate that protein films have limited ability to block moisture, are prone to dissolving, and their mechanical strength can be easily damaged [15]. Also, plant proteins exhibit relatively low gel strength due to their nature as supramolecular structures with a globular conformation, thereby necessitating enhancements in their gelation properties. In essence, improved gelation imparts advantages to the films, such as enhanced mechanical properties and superior gas barrier capabilities [16,17]. Ultimately, no films prepared with plant proteins alone meet the requirements of food preservation, including superior mechanical performance, effective barrier properties, and other critical factors. The development of edible films derived from plant proteins with improved physical and chemical properties has become a matter of concern. Therefore, various methodologies have been employed to develop plant protein-based films that demonstrate enhanced structural stability, alongside improved mechanical and barrier properties [12]. In this context, one approach to enhance their characteristic properties is by incorporating polysaccharides and lipid structures into the related systems. Edible coatings were created using soy protein isolate and gelatin, and their effects on preventing the softening of apricots were investigated. This blend coating (soy protein isolate–gelatin) demonstrated superior protective performance on fruits compared to soy protein and gelatin coatings alone [18]. Similarly, various films were produced using apple pectin, carboxymethyl cellulose, egg white proteins, and tartaric acid to enhance the low mechanical strength and poor water resistance of wheat gluten films. The carboxymethyl cellulose–wheat gluten complex films exhibited excellent thermal stability, while the addition of pectin improved the morphological results of the films [19]. Acetic esters of mono- and diglycerides, along with beeswax, were used to create edible films made from wheat gluten. Upon reviewing the findings, it was noted that hardness decreased, extensibility increased, and water affinity lessened [20]. However, these factors also present certain challenges. For example, the incorporation of lipids enhances the flexibility of the films; however, excessive addition leads to undesirable flexibility and oxidation within the structure. Furthermore, due to the hydrophobic nature of lipids, achieving a homogeneous distribution within the film systems may be difficult in the film systems. A similar situation applies to polysaccharides. The usage of polysaccharides is constrained because of their high-water vapor permeability and fragility [21]. Additionally, these combinations can disrupt the protein structure function within the film system and, in some cases, entirely remove the protein’s primary structural role in the film. This complicates classifying the film as a “pure protein film” and introduces ambiguity in defining the film system. Consequently, although incorporating auxiliary components like carbohydrates or lipids into protein-based film systems improves targeted mechanical or barrier properties, the disruption of the original protein structure constitutes a significant downside of this method [22].

In this study, quercetin, a powerful polyphenol, was incorporated into the film system. However, even though polysaccharide and lipid-based systems are used to deliver bioactive compounds, these carbohydrate structures can lead to negative nutritional effects, especially when eaten in excess. Currently, using protein-based edible films to deliver functional compounds, such as quercetin, could be considered a significant advancement, offering a healthier option for functional packaging. Therefore, to fully accomplish the objective, it is essential to design such edible film systems with proteins alone. Reports suggest that the functionality of plant proteins enhances when they form complexes with animal proteins, especially considering that films composed of a single protein are ineffective [23].

The properties of protein films are influenced by ionic and disulfide bonds. The strength of the interactions between protein chains impacts the mechanical and barrier properties of the films [24]. The capacity to create suitable films through protein–protein interaction largely depends on the protein concentration. Low concentrations do not provide enough intermolecular interactions, while high concentrations lead to poor protein distribution [25]. Thus, using appropriate ratios is essential to create a stable structure in protein–protein interaction. In the present study, whey protein was utilized to enhance the film-forming properties of chickpea protein isolates and promote the development of protein-based films. When datasets in the scientific literature are checked, film systems incorporating chickpea–whey protein composites have not been previously created. This study introduced an innovative method to improve the film-forming ability by incorporating varying amounts of whey protein into chickpea protein. The aim was to enhance the performance of chickpea protein-based films by leveraging the benefits of whey protein, including its high solubility, flexibility, and bonding capacity. This combination introduces an innovative methodology to produce more durable and functional biofilms by demonstrating the synergistic effect of both plant and animal proteins. Therefore, these findings could significantly contribute to the development of hybrid protein-based materials with improved properties for use in food packaging and edible films. Furthermore, this research presents an innovative film formulation by integrating the natural antioxidant quercetin into an edible film composed of chickpea protein and whey protein. Although the film-forming properties of each protein have been studied separately in the literature, the properties of the films created by adding quercetin to various protein ratios were evaluated in detail for the first time. Additionally, using these films as coatings on strawberries, a real food model, and demonstrating their protective effects on shelf life highlight the practical importance of the study. In this context, the study possesses the potential to contribute to the literature by advancing both biodegradable active packaging and functional food preservation strategies.

In this study, sustainable, environmentally friendly, and high functionality protein–protein edible film systems were developed. For this purpose, the ratios of chickpea protein and whey protein used in film production were optimized. The morphological, physicochemical, barrier, and mechanical properties of the packaging material were examined. Additionally, the release properties in various food simulants (aqueous, oily foods, and alkaline environments) were analyzed. Finally, the protective effects of film-forming solutions on the edible coating of strawberry fruit were also evaluated.

## 2. Materials and Methods

### 2.1. Materials

Chickpea seeds were sourced from the local market (Sanliurfa, Türkiye). Whey protein concentrate (protein content: 70%) was acquired from Kalipso Chem (Istanbul, Türkiye). Glycerol and ethanol were obtained from Merck (Darmstadt, Germany). Quercetin was purchased from Sigma-Aldrich Co., Ltd. (St. Louis, MO, USA). All other chemicals used were of analytical grade.

### 2.2. Production of Chickpea Protein

Chickpea proteins were fabricated based on a prior study with minor modifications [26]. Chickpeas were ground and sifted using 80 mesh. Chickpea powder and hexane were mixed at ambient temperature for 3 h to remove the oil (to avoid interactions between lipids and proteins and to increase protein purity). Defatted chickpea flour was combined with 1:10 (*w*/*v*) distilled water and stirred for 30 min. Next, the pH was adjusted to 9.0, stirred for 2 h, and centrifuged (4000 rpm, 10 min) (NF 1200R, Nüve, Ankara, Türkiye) to collect the supernatant. The supernatant was adjusted to pH 4.5 and stored at +4 °C overnight to precipitate the protein. Then, the precipitated proteins were recovered by centrifugation at 4000 rpm for 10 min. Finally, the recovered proteins were dried by lyophilization (Coolermed, Manisa, Türkiye). The protein content of chickpeas was measured using the Bradford method, yielding a value of 73.34% [27]. The proteins produced were used to make edible films.

### 2.3. Fabrication of Edible Film

Chickpea protein-derived films were produced using the casting method with slight modifications [28]. The formulations for protein films are provided in Table 1. In summary, chickpea protein alone and mixtures of chickpea–whey protein were dispersed in distilled water (5 g/100 mL) to prepare film-forming solutions (pH: 8.0). Next, this solution was continuously stirred and heated to 65 °C (in a water bath) for 1 h to denature the proteins. After cooling this blend, glycerol (as a plasticizing agent) and quercetin (which was dissolved in ethanol) were added and mixed with a magnetic stirrer (note: the quercetin ratio was selected based on a previous study [29]). Then, the film solution was degassed using an ultrasonic degassing process to remove bubbles. Finally, the film forming solutions (5 mL) were added to the Petri dishes and air-dried (without sunlight) at 25 °C for 48 h. Films were named CF (chickpea protein alone film), CWF1 (film containing 90% chickpea protein and 10% whey protein), CWF2 (film containing 80% chickpea protein and 20% whey protein), CWF3 (film containing 70% chickpea protein and 30% whey protein), CWF4 (film containing 60% chickpea protein and 40% whey protein), and CWF5 (film containing 50% chickpea protein and 50% whey protein).

### 2.4. Analyses

#### 2.4.1. Amino Acid Profile of Proteins

The amino acid (AA) profile of proteins was analyzed based on a prior study [30]. The AA composition of proteins was evaluated using an LC-MS-8040 device (Shimadzu, Kyoto, Japan). Analysis was conducted after samples were prepared following the commercial Jasem amino acid kit (SEM Laboratory Devices Industrial Marketing and Tarde, Istanbul, Türkiye) protocol. Solvents A (formic acid: 0.05% (*v*/*v*)) and B (acetonitrile) served as the mobile phase (A:B, 30:70, *v*/*v*). The column temperature, flow rate, and injection volume were set at 30.0 °C, 0.07 μL/min, and 40 μL, respectively. The detection and evaluation of amino acids were carried out using an Agilent C18 XDB column (3.5 μm 3 × 150 mm). The data obtained were processed using Shimadzu LabSolutions Connect software (version 5.80), and the quantitative results were obtained.

#### 2.4.2. Fourier Transform Infrared Spectroscopy (FTIR)

Infrared spectra of protein films were obtained using an FTIR spectrometer (IRTracer-100, Shimadzu Co., Kyoto, Japan) by placing the measuring probe directly on the film surface. All samples were scanned at 4 cm^−1^ resolution, with 32 scans over a wavelength range of 500–4000 cm^−1^ [31]. The background spectrum was recorded before each measurement.

#### 2.4.3. Scanning Electron Microscopy (SEM)

The morphology of the film was based on a previous study [32]. The microstructures (surface and cross-section) of films were scanned using SEM (Zeiss Sigma 300 Field Emission Sem, Oberkochen, Germany) at 10.0 kV voltage. Before being visualized, the films (gold-coated and cryo-fractured) were affixed to conductive tape. Additionally, the films were broken before capturing the cross-sectional images. Then, the film images were captured through proper scanning.

#### 2.4.4. Moisture Content

The moisture content of edible natural films was evaluated by measuring water loss from the initial weight. Before analysis, the samples were stored in a desiccator to prevent their exposure to ambient humidity. First, the films were cut into 2 cm × 2 cm pieces, and their weight was recorded (*m*1). Then, the samples were placed in a drying oven at 105 °C and dried until they reached an equilibrium weight (*m*2) [33]. The *moisture content* was calculated using the following equation (Equation (1)):

(1) $$Moisture\;content\left(\%\right)=\frac{m1-m2}{m1}\times 100$$

#### 2.4.5. Thickness

The thickness of the films was measured using a digital caliper with an accuracy of 0.01 mm (D&W, Model DW1KDS15, Hersbruck, Germany). In this context, measurements were randomly taken from different regions (*n* = 10) of the films, including the center, edge, and cross areas, and the thickness was determined by averaging these values [34].

#### 2.4.6. Color and Opacity

The color parameters (L*, a*, and b*) of the films were determined via a HunterLab calorimeter (Color Quest^®^ XE, Reston, VA, USA). The films were correctly placed in the reading section of the colorimeter. The L*, a*, b* values were calculated using the device EasyMatch QC software (version 4.90) under D65 illuminant and 10 observer angles. Here, L*, a*, and b* values represent lightness, redness-greenness, and yellowness-blueness, respectively [35].

The opacity parameters of protein films were determined using a spectrophotometer (Model UV-1280, Shimadzu Co.). In this context, the films were cut into 1 × 4 cm^2^ pieces and fixed to the wall of the spectrophotometer cuvette, and the absorbance value at 600 nm was measured. This value was calculated by dividing the absorbance by the thickness (mm) [36].

#### 2.4.7. Swelling Ratio and Solubility

The swelling ratio and solubility of the films were measured following a prior method [37]. Each film sample was stored in a desiccator before analysis and then cut into 2 cm × 2 cm pieces. Then, the film samples were weighed (*m*1) and dried at 105 °C for 24 h (*m*2). The dried films were placed in 20 mL of distilled water, 50% ethanol, and 95% ethanol and after 24 h; excess solution was removed using filter paper. Next, the swollen films were weighed, and their weights (*m*3) were recorded. Afterwards, these undissolved films were dried under the same conditions (105 °C, 24 h), and the final weight (*m*4) was noted. The *swelling ratio* and *solubility* were calculated using the following equations (Equations (2) and (3)):

(2) $$Swelling\;ratio\left(\%\right)=\frac{m3-m2}{m2}\times 100$$

(3) $$Solubility\left(\%\right)=\frac{m4-m2}{m1}\times 100$$

#### 2.4.8. Water Vapor Permeability

Water vapor permeability (WVP) was assessed based on a previous study [38]. Containers with silica gel (0% RH) were capped with films (inner diameter: 5 cm). The containers were placed in a desiccator containing distilled water (25 °C, 100% RH) and left for 24 h. Next, containers were kept for 24 h and weighed every 60 min. The equation below (Equation (4)) was utilized to calculate *WVP*:

(4) $$WVP=\frac{w\times a}{t\times A\times \Delta P}$$

*w*: change in weight (g); *a*: film thickness (mm); *t*: time (h); *A*: surface area of film (m^2^); Δ*P*: pressure difference in silica gel with distilled water (kPa)

#### 2.4.9. Oxygen Permeability

Oxygen barrier permeability was determined using a prior method with minor modifications [39]. Fifteen mL of sunflower oil was transferred to 50 mL beakers, and their openings were covered with films. The beakers were kept at room temperature for one month (30 days), and peroxide values were measured every 5 days using the sodium thiosulfate method. Peroxide analyses conducted at regular intervals during the storage period were used to assess the barrier performance of the films against oxidation. Peroxide values were reported in meq O_2_/kg.

#### 2.4.10. Mechanical Behaviors

The mechanical properties of the films, including tensile strength (TS) and elongation at break (EAB), were determined via a texture analyzer (TA-XT32, Stable Micro System, Godalming, UK) equipped with an AT/G mini tensile grip. The test speed, post-test speed, distance, and starting force (break sensitivity) values required to initiate the test, as determined by the analysis parameters, were 1 mm/s, 10 mm/s, 50 mm, and 10 g, respectively. Films were cut in accordance with the film pulling apparatus, and 3 measurements were made for each sample. The films positioned between the tensile apparatus underwent analysis, and the textural properties were automatically measured by the instrument’s Exponent software (version 6.1.10.0) [32].

#### 2.4.11. Release Profile in Food Simulants for Quercetin

The release behavior of the films was evaluated with minor modifications in an earlier study [40]. The quercetin released from the films was determined in three aqueous food simulators including distilled water, 50% ethanol, and 95% ethanol. These simulators represent aqueous, fatty foods, and alkaline mediums. Films cut into equal sizes (3 × 3 cm) were placed in food simulators and kept at an ambient temperature of 72 h. Aliquots were taken at 1, 3, 6, 9, and 12 h after the initial measurement, and then every 6 h thereafter. For measurements, 2 mL of release solution was drawn by a pipette (an equal volume of medium solution was added at each intake). The quercetin ratio was measured via the calibration curve. To achieve this, a specific amount of quercetin was prepared and then diluted to various concentrations. The calibration curve was generated by measuring the absorbance of the solution at 371 nm. The release rate was calculated based on the standard curve [41].

#### 2.4.12. Strawberry Preservation Using Edible Coating Films

Fresh strawberries were selected to be at the same level of ripeness, physically whole, and of similar size. The selected strawberries were randomly split into groups. No treatment was given to the control group. Various protein-based film solutions were prepared for the coating process, and about 50 mL of solution was used for each coating group. Each strawberry was fully immersed in its respective coating solution for 1 min. Afterwards, they were drained and allowed to dry at room temperature (3 h). Uncoated and coated samples were kept at room temperature for eight days. Changes in the samples were documented with a digital camera, and analyses were conducted every two days [42].

##### pH and Titratable Acidity

For the pH value of strawberries, 20 g of sample was weighed and homogenized. Then, a measurement was made using a pH meter (Woonsocket, RI, USA) [43]. For the titration of acidity, 5 g of homogenized sample was weighed, and 50 mL of distilled water was added and allowed to sit for 30 min. Then, 20 mL of the filtrate was taken and titrated with 0.1 mol/L NaOH to a pH of 8.1 [37]. The *titratable acidity* was calculated according to the following equation (Equation (5)):

(5) $$Titratable\;acidity\left(\%citric\;acid\right)=\frac{V\times 0.1\times 0.064\times V0}{m\times V1}\times 100$$

*V*: volume of NaOH expenditure; *V*0: total solution volume; m: mass of filtrate; *V*1: the volume of filtrate (20 mL); 0.064: equivalent weight of citric acid

##### Weight Loss and Hardness

The *weight loss* of coated and uncoated strawberries was calculated using the equation below (Equation (6)):

(6) $$Weight\;loss\left(\%\right)=\frac{Wi-Wf}{Wi}$$

*Wi*: initial mass of sample; *Wf*: final weight of sample at various storage intervals

The hardness of strawberries was determined using a texture analyzer equipped with a cylindrical probe (P/5). The samples were placed on the analyzer’s platform, and the measurements were carried out automatically through the instrument’s Exponent software (version 6.1.10.0). The test was conducted at a speed of 10 mm/min, and the hardness values were recorded in grams (g) [44].

#### 2.4.13. Statistical Analysis

All experiments were exhibited as the mean ± standard deviation (*n* = 3). Figures were generated via OriginPro 2021b (Origin Lab Inc., Northampton, MA, USA). One-way analysis of variance (ANOVA) and Tukey’s multiple comparison tests were employed for differences between mean values at a significance level of *p* ≤ 0.05. Statistical datasets were analyzed by using SPSS software (version 22.0; SPSS Inc., Chicago, IL, USA).

## 3. Results and Discussions

### 3.1. Amino Acid Composition

Table 2 presents the amino acid composition of chickpea protein and whey protein. The essential amino acid content of chickpea protein was 16.48%; this value was 8.09% for whey protein. Lysine, phenylalanine, and tryptophan were the key essential amino acids in chickpea protein. The prominent essential amino acids in whey protein were leucine, lysine, methionine, and valine. Significant differences were noted in non-essential amino acids. For whey protein, alanine, anserine, and sarcosine were predominant, while for chickpea protein, arginine, and glutamic acid were the predominant non-essential acids.

### 3.2. FTIR Spectroscopy

**Figure 1:**
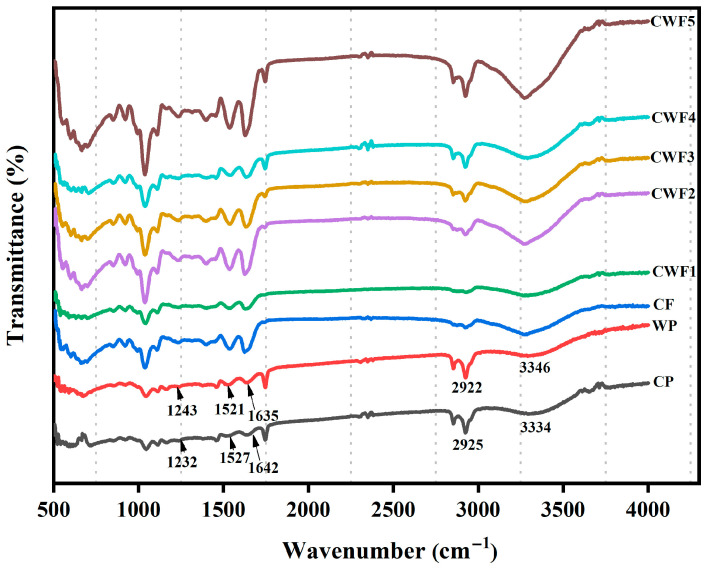
FTIR spectra of chickpea protein-derived films. CP (chickpea protein), WP (whey protein); CF (chickpea protein alone film), CWF1 (film containing 90% chickpea protein and 10% whey protein), CWF2 (film containing 80% chickpea protein and 20% whey protein), CWF3 (film containing 70% chickpea protein and 30% whey protein), CWF4 (film containing 60% chickpea protein and 40% whey protein), and CWF5 (film containing 50% chickpea protein and 50% whey protein).

FTIR spectroscopy was performed to analyze the chemical structures of the raw materials forming the film complexes and to detect potential intra- and intermolecular interactions within them. The FTIR spectra of raw materials and films are demonstrated in Figure 1. The characteristic absorption bands observed for chickpea protein (CP) and whey protein (WP) appeared at 3334–3346, 2925–2922, 1642–1635, 1527–1521, and 1232–1243 cm^−1^. These bands correspond to the typical amide regions: amide A (3000–3500 cm^−1^), amide B (2850–2980 cm^−1^), amide I (1600–1700 cm^−1^), amide II (1500–1550 cm^−1^), and amide III (1200–1300 cm^−1^), respectively. Specifically, the amide A band is attributed to O–H and N–H stretching vibrations, which are typically involved in hydrogen bonding and intramolecular interactions [45]. The amide B band reflects the stretching vibrations of C–H and NH_2_ groups; amide I is primarily associated with the C=O stretching of peptide bonds, whereas amide II corresponds to the combination of C–N stretching and N–H bending vibrations. The amide III region is linked to C–N and C–C stretching as well as N–H bending. In addition to these protein-specific bands, a distinct absorption region between 1000 and 1100 cm^−1^ was identified, indicating the presence of glycerol used as a plasticizer in the film matrix [46].

Incorporating multiple polymers into film formulation often alters spectroscopic features due to physical interactions and possible chemical bonding between components [47]. In this case, adding whey proteins (WP) to chickpea protein (CP)-based film matrices caused noticeable changes in the FTIR absorption bands, especially in the main amide regions of the protein structures. One of the most notable changes was observed in the amide I region. In composite CP–WP films, the amide I band appeared between 1629 and 1637 cm^−1^, showing a clear blueshift compared to the control film (CF), where the corresponding peak was observed at 1624 cm^−1^. This spectral shift is commonly attributed to electrostatic interactions occurring between the positively charged amino groups (–NH_3_^+^) and negatively charged carboxyl groups (–COO^−^), which alter the local chemical environment [48]. Moreover, since the amide I band primarily originates from the C=O stretching vibrations of the peptide backbone, its displacement suggests conformational changes in the secondary structure, particularly transitions in α-helix and β-sheet motifs [49]. Parallel to these observations, the amide II region—linked to N–H bending and C–N stretching—also showed a significant shift after WP incorporation, further indicating structural changes at the molecular level. Although changes in the amide III band were more subtle, the observed variations still suggested modifications in protein folding and interchain interactions within the film matrix. Overall, these changes suggest that adding WP promotes the development of more organized β-sheet structures, probably due to increased hydrogen bonding and electrostatic interactions during film formation [48,50]. These molecular interactions are believed to involve side chains of hydrophilic amino acids such as glutamic acid, asparagine, and serine, which facilitate the observed spectroscopic changes. Furthermore, the interaction between aromatic residues (e.g., tryptophan and phenylalanine) and hydrophobic amino acids in chickpea protein, along with nonpolar residues like alanine, valine, and isoleucine in whey protein, may lead to hydrophobic clustering within the film [51]. This phenomenon was supported by the broadening of the –OH/NH stretching band in the 3334–3346 cm^−1^ range, indicating the formation of hydrogen bonds between hydrophilic groups of WP and the acidic or basic side chains in CP [52]. These results were consistent with earlier research, which has shown that nanoparticles cause structural changes in zein and gelatin matrices [53].

### 3.3. Visual and SEM Appearance

**Figure 2:**
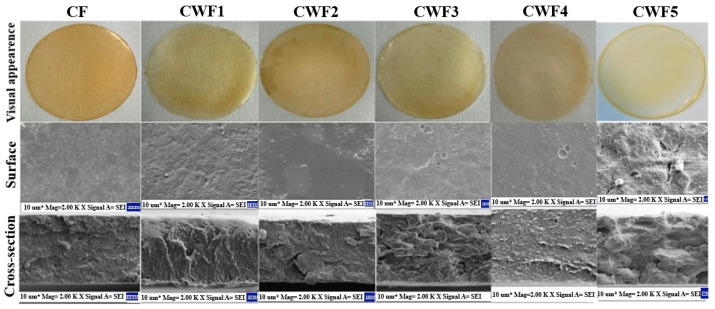
Visual images, surface, and cross micrographs (SEM) of chickpea protein-derived films. CF (chickpea protein alone film), CWF1 (film containing 90% chickpea protein and 10% whey protein), CWF2 (film containing 80% chickpea protein and 20% whey protein), CWF3 (film containing 70% chickpea protein and 30% whey protein), CWF4 (film containing 60% chickpea protein and 40% whey protein), and CWF5 (film containing 50% chickpea protein and 50% whey protein).

This section examines the morphology of chickpea protein-derived films containing whey protein to visualize the whey protein distribution pattern in the film. Visual images of chickpea–whey protein mixture film systems and findings regarding SEM (surface and cross-section) micrographs are shown in Figure 2. Actual images of the films indicate that there is no breakage or tearing after drying. In terms of visual appearance, CF (chickpea protein alone) and CWF4 (60:40 chickpea–whey protein ratio) films exhibited a uniform color and structure, while CWF1, CWF2, CWF3, and CWF5 showed visible heterogeneity with areas of differing coloration. This variation may be attributed to insufficient or excessive whey protein levels, which disrupt optimal protein–protein interactions and hinder homogeneous matrix formation [25]. Surface SEM images showed that the CF film had an irregular texture with visible insoluble particles. The addition of whey protein initially increased surface roughness and irregularity (CWF1–CWF3), likely due to aggregation and incomplete dispersion. However, at 40% whey protein (CWF4), the surface morphology became more uniform and compact, indicating stronger protein–protein interactions and improved matrix cohesion. This is supported by the film’s lower solubility values, suggesting enhanced structural integrity. When whey protein content exceeded 40% (CWF5), the surface again became heterogeneous and disrupted, potentially due to phase separation or oversaturation of protein interactions, leading to structural instability. Similar trends were observed in other protein-based film systems, such as soy protein isolate–grasshopper protein–cinnamaldehyde mixtures, where excessive protein disrupted uniformity [54], and in ternary alginate/carboxymethyl cellulose/starch films, where high potato starch content led to morphological deformations [55]. Cross-sectional images revealed that CF and CWF1 showed a relatively rough network structure, while CFW4 displayed a more homogeneous network structure. In contrast, CWF2, CWF3, and CWF5 films exhibited a heterogeneous, rough, and scattered network structure.

### 3.4. Physicochemical Properties, Solubility, and Swelling Ratio

The effects of different whey protein concentrations on the moisture content (MC) and thickness of chickpea protein-derived films are presented in Table 3. MC exhibited significant changes with an increase in whey protein content. Namely, the highest water content was found in CWF5 (20.18%), followed by CWF3 (18.01%), CWF1 (17.99%), CF (17.86%), CWF2 (17.76%), and CWF4 (16.12%). This can be attributed to the phenomena of hydrogen bonds and hydrophobic interactions among amino acids in proteins, which result in the formation of a strong network structure due to protein–protein interaction [56]. As a result, films formed with an appropriate chickpea–whey protein ratio can reduce the moisture content by reducing the polymer–water binding areas. In the study examining the effects of octenylsuccination (OSA) of pullulan (PU) modification on pullulan–chickpea protein composite films, a reduction in moisture content was reported due to the increased concentration of chickpea protein (CPI) [57].

It has been reported that the ideal thickness of the films should be less than ≤0.25 mm (ASTM international standard) [58]. The findings for the thickness are presented in Table 3. The thickness varied from 0.10 mm to 0.15 mm. This value for CWF1 (0.10 mm) and CWF2 (0.11 mm) was the same or close to control (0.10 mm). Shifting whey protein concentration to 30% in the system led to a partial increase in thickness and the value was 0.12 mm for CWF2 (*p* < 0.05). On other hand, raising the whey protein ratio to 40% resulted in a reduction in thickness. A noticeable increase in this value was observed by incorporating high concentrations (50%) of whey proteins into this system. The highest thickness rate was in CWF5 (0.15 mm). A similar situation has been reported in pullulan–chickpea protein isolate composite films, where thickness values augment as the chickpea protein ratio increases [57]. In the characterization study of alginate/carboxymethyl cellulose/starch triple blend film for packaging applications, it was stated that the thickness values decreased with increasing potato starch ratio [55]. The decrease in thickness value against increasing whey protein ratio can reduce the free volume and film thickness between the polymer matrix by making the mixture structure more compact due to strong bonds formed with protein–protein interaction [59]. In a study examining the impact of humidity on the thermal and barrier properties of EVOH films made from ethylene vinyl alcohol, a film thickness of 55 µm was reported [60]. Similarly, PVC films were created using polypyrrole (PPy) and barium hexaferrite (BaFe), with a thickness set at 0.5 mm [61].

The consumer assesses the quality of the product based on the appearance of the packaging. Natural-looking films are more reassuring. In summary, conducting color analysis on films is essential for establishing trust and fostering consumer preference. In this context, color attributes, namely L*, a*, and b* of films were investigated, and the related findings are illustrated in Table 3. Whey protein concentration led to a gradual increase in the L* values of films (CF: 82.08; CWF1: 84.15; CWF2:84.73; CWF3: 85.51; CWF4: 85.94; CWF5: 86.32) (*p* < 0.05). This indicates an increase in film lightness. Whey protein is white, which increases the white value and saturation of films; thus, the lightness (L*) value rises. The visual images of the films also support this situation. The green color (a*) and yellow color (b*) values of chickpea protein-derived films exhibited similar trends. To put it another way, both a* (CF: −0.28; CWF1: −0.34; CWF2: −0.44; CWF3: −0.66; CWF4: −0.70; CWF5: −0.76) and b* (CF: 28.37; CWF1: 27.00; CWF2: 26.07; CWF3: 25.54; CWF4: 24.89; CWF5: 24.35) values showed a decrease. In the study on pullulan–chickpea protein composite films, L*, a*, and b* values of chickpea protein films were reported as 78.07, −2.64, and 12.57, respectively [57].

In food coating applications, films with high opacity can obstruct light transmission, thereby minimizing light oxidation of foods [62]. The opacity values of chickpea protein-derived film systems are presented in Table 3. The opacity values of the films were determined to be in the range of 1.73–2.55. The opacity value of CF (1.73 Abs/mm) was lower than those of films containing whey protein. These values for CWF1, CWF2, and CWF3 were 1.85, 1.76, and 1.74 Abs/mm. Continuing to increase whey protein concentration in films resulted in an increase in opacity (CWF4: 1.93 Abs/mm and CWF5: 2.55 Abs/mm) (*p* < 0.05). Similar findings were also recorded in the literature. Addition of gum tragacanth at different rates to whey protein films increased the opacity values [63]. In another study, it was reported that opacity values increased with the addition of carboxymethyl cellulose (CMC), egg white proteins (EWP) and tartaric acid (TA) to wheat gluten protein films [19]. A study on petroleum-based films involved producing composite films with various ratios of a poly-ɛ-caprolactone (PCL) and polyvinyl chloride (PVC) mixture. Opacity values for these films ranged from 0.61 to 2.41 (Abs/mm) [64]. Similarly, the opacity value of T/PP—polyethylene terephthalate/polypropylene film—was 1.21 Abs/mm [65].

The swelling ratio of the film systems was determined in 3 different environments, water, 50% ethanol, and 95% ethanol, and the relevant findings are presented in Table 4. In the water phase, the highest swelling was observed in CWF4 (282.02%). This was followed by CWF3 (266.32%), CWF2 (188.79%), and CWF1 (186.09%), in that order. The swelling ratio of CF was 171.85% in the water phase. As the proportion of whey protein increased, the swelling ratio of the films rose, reaching its highest value in CWF4 (283.02%), followed by CWF3 (266.32%), CWF2 (188.79%), and CWF1 (186.09%).

In the study of soy protein–gelatin blend films, it was reported that the degree of swelling of the film increased with the amount of gelatin content added [66]. In contrast, excessive addition of whey protein (CWF5: 164.16%) negatively affected the swelling degree of chickpea–whey films. Adding whey protein at a specific rate may ensure balanced protein interactions. Consequently, this leads to a denser, more compact network structure that enhances water retention capacity [67]. Additionally, high protein levels negatively impacted the swelling rate. This decrease can be attributed to phase separation, intense cross-linking, or deterioration in matrix homogeneity caused by high whey content. In a study, it was reported that zein addition above 5% could cause more interaction, which could harm the cross-linking reaction and lead to undesirable outcomes [68]. The results were also in agreement with the SEM images. In 50% ethanol medium, swelling values were generally slightly lower than in water medium, but similar trends were observed among the samples. CWF4 and CWF3 again demonstrated the highest swelling capacity, while CF and CWF5 showed the lowest values. All samples showed a significant reduction in swelling in a 95% ethanol medium. The least swelling was seen in CF (52.41%) and CWF5 (54.45%), while the greatest swelling was in CWF3 (70.16%) and CWF4 (66.06%). This indicates that high ethanol levels restrict water absorption and matrix swelling by enhancing hydrophobic interactions within the protein matrix, thereby reducing film–water interactions [69,70]. The swelling abilities of gelatin films with varying amounts of free/nano encapsulated tea polyphenols were tested in 50% ethanol and 95% ethanol solutions. It was reported that swelling was higher in 50% ethanol than in 95% ethanol [70].

The solubility of films is closely associated with the degree of cross-linking in the polymer network. A denser network structure limits the penetration of water molecules and prevents film dissolution by reducing the number of available hydrophilic sites [71]. Normally, low water loss is a basic and mandatory condition for food packaging films for the overall integrity of the film and the signs of its waterproofness [69]. The solubility of films was analyzed in 3 different phases, water, 50% ethanol, and 95% ethanol, and the related results are illustrated in Table 4. The solubility in water, 50% ethanol, and 95% ethanol phases of the film produced from chickpea protein alone was 73.05, 69.97, and 35.24%, respectively. Upon the incorporation of whey protein, the solubility values decreased across all solvent systems, with the lowest values observed for CWF4. This trend continued up to 40% whey content; beyond that (CWF5), the effect reversed. Similar findings were reported by Omar-Aziz et al., 2021 [57]. They reported a decrease in solubility values after the modification process in chickpea protein–pullulan composite films. Similar results were observed in gelatin–zein films, where a higher zein ratio led to lower solubility [72]. These findings can be attributed to the increase in the degree of crosslinking, which can prevent moisture penetration. On the other hand, crosslinking also reduces the hydrophilic groups of polymers [73,74,75]. As a result, the solubility values of protein–protein containing films decrease due to the formation of strong intermolecular bonds. However, when the effect of different solvent environments was examined, the solubility was highest in water, followed by 50% and 95% ethanol. The lowest solubility was observed in 95% ethanol. This depends on the polarity of the solvent environment. Namely, proteins exhibit water solubility due to their hydrophilic structure [76]. Kim et al. (2021) [77] extracted proteins from *Protaetia brevitarsis* larvae and assessed their solubilization using two different methods. The protein percentages in water and ethanol conditions were 66.80% and 32.14%.

### 3.5. Water Vapor Permeability and Oxygen Barrier Attributes of Chickpea Protein-Derived Films

**Figure 3:**
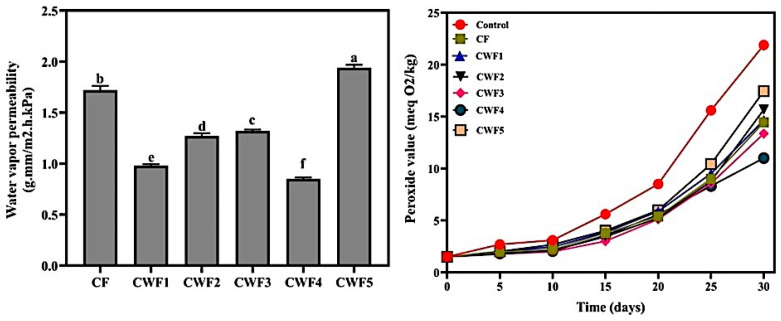
Water vapor permeability and oxygen barrier properties of chickpea protein-derived films. All results express mean ±  SD (standard deviation). ^a–f^ Lowercase letters in the same graph present statistical differences (*p* < 0.05). CF (chickpea protein alone film), CWF1 (film containing 90% chickpea protein and 10% whey protein), CWF2 (film containing 80% chickpea protein and 20% whey protein), CWF3 (film containing 70% chickpea protein and 30% whey protein), CWF4 (film containing 60% chickpea protein and 40% whey protein), and CWF5 (film containing 50% chickpea protein and 50% whey protein).

The measured water vapor permeability (WVP) and oxygen barrier attributes of chickpea protein-derived films are illustrated in Figure 3. Low WVP values are crucial in food packaging for extending the shelf life of products [78]. Based on findings from Figure 3, changing the concentration of whey protein affected the WVP of films. The WVP of the CF film was 1.72 g.mm/m^2^.h.kPa. The WVP of films decreased until the whey protein ratio (CWF3: 1.32; CWF2: 1.27; CWF1: 0.98, and CWF4: 0.85 g.mm/m^2^.h.kPa) reached 40%. Improvements in the WVP were also reported in a previous study. WVP values decreased due to the increased ratio of zein (0% to 50%) in caseinate/zein films [79]. These decreases in WVP of the films can be attributed to increased protein–protein interactions, which effectively reduced the free volume in the film structure, thereby creating fewer hydrophilic voids for water binding [80]. As demonstrated by the SEM images, the formation of a compact network leads to excellent barrier properties. The uniform distribution of polymer matrices leads to the formation of a compact film structure through protein–protein hydrogen bonds, which in turn slows down the diffusion of water vapor through the film. In other words, this situation increased the tortuosity and complexity of the water molecules’ diffusion path, enhancing the film’s hydrophobicity and reducing its WVP [81]. Therefore, CWF4, which had a uniform appearance and a dense film matrix, showed the minimum WVP. This finding supports the idea that a well-structured and compact polymer network effectively restricts water vapor diffusion. Conversely, increasing the whey protein concentration beyond 40% (as in CWF5) resulted in a higher WVP (1.94 g.mm/m^2^.h.kPa). This negative effect is probably caused by a disruption in the film’s structural integrity at high whey levels. Excessive whey protein can lead to protein aggregation, phase separation, or too much cross-linking, all of which can create free spaces and tiny irregularities in the film matrix, making water vapor pass through more easily. A similar situation was observed in gelatin–zein composite films. Adding more zein resulted in larger zein particles, which increased the space between chains of gelatin and zein, weakening the WVP functionality [68].

Many food products can deteriorate easily because they contain lipids in their structure. Therefore, a sensible approach is to develop coating materials with enhanced oxygen barrier properties to extend shelf life and preserve quality [82]. In this section, sunflower oil was covered with chickpea protein-derived films, and the oxygen barrier properties of these films were monitored by observing the alterations in the peroxide value (PV) of the oil over 30 days at ambient temperature. The relevant datasets are presented in Figure 3. The initial PV of sunflower oil used in this study, in terms of oxygen barrier properties, was measured as 1.50 meq O_2_/kg. No significant increase was observed in the value of these films during the first 5 days; however, it was statistically significant (*p* < 0.05). By the 5th day, the PV of the uncoated (control) sample (2.70 meq O_2_/kg) was higher than that of the film-applied samples, which ranged from 1.76 to 2.01 meq O_2_/kg. A similar trend persisted through the 10th day, and the increase was minimal. Meanwhile, on the 15th day, the lowest PV was recorded as 3.01 meq O_2_/kg (CWF3), and the highest as 5.6 meq O_2_/kg (control) (*p* < 0.05). However, on the 25th day, a significant change occurred in the PV of the samples and continued until the end of storage. On this day (25th), the PV of the samples ranged between 8.32 and 15.60 meq O_2_/kg, and a statistically significant difference was detected among the samples (*p* < 0.05). At the end of storage, the lowest PV was observed for CWF4 (11.01 meq O_2_/kg), followed by CWF3 (13.36 meq O_2_/kg), CF (14.45 meq O_2_/kg), CWF1 (14.65 meq O_2_/kg), CWF2 (15.67 meq O_2_/kg), CWF5 (17.45 meq O_2_/kg), and the control (uncoated) (21.89 meq O_2_/kg) (*p* < 0.05). Moreover, the changes in the PVs of the samples during storage were statistically significant (*p* < 0.05). Oxidation in the samples continued gradually and slowly until the 20th day. The increase in the PVs of all samples by the end of storage was significant (*p* < 0.05). The superior performance of CWF4 can be attributed to improved intermolecular interactions—such as hydrogen bonding and hydrophobic interactions—between chickpea and whey proteins, which enhanced film uniformity and decreased oxygen permeability [83]. FTIR results confirmed the formation of these structural interactions. Conversely, films with 50% whey protein (CWF5) showed reduced performance due to structural heterogeneity, likely caused by phase separation or excessive cross-linking. This observation was supported by SEM images. Similar findings were revealed in a different study in the literature. Films produced with whey protein isolate, and psyllium seed gum (1:1 *w*/*w*) have been reported to possess the highest oxygen barrier [84].

### 3.6. Mechanical Properties of Chickpea Protein-Derived Films

**Figure 4:**
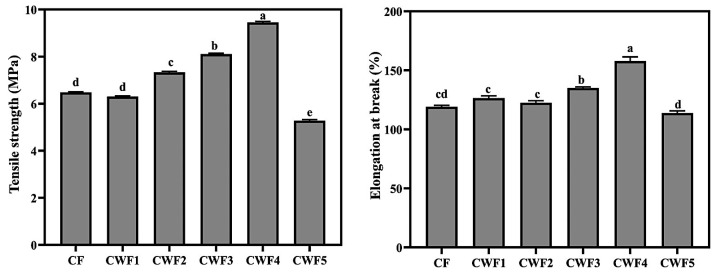
Tensile strength (TS) and elongation at break (EB) of chickpea protein-derived films. All results express mean ±  SD (standard deviation). ^a–e^ Lowercase letters in the same graph present statistical differences (*p* < 0.05). CF (chickpea protein alone film), CWF1 (film containing 90% chickpea protein and 10% whey protein), CWF2 (film containing 80% chickpea protein and 20% whey protein), CWF3 (film containing 70% chickpea protein and 30% whey protein), CWF4 (film containing 60% chickpea protein and 40% whey protein), and CWF5 (film containing 50% chickpea protein and 50% whey protein).

Mechanical strength and flexibility are vital properties for edible films to withstand conditions during food processing, packaging, and storage [85]. Tensile strength (TS) and elongation at break (EAB) of the chickpea protein-derived films are demonstrated in Figure 4. Including 10% whey protein (CWF1) had a minimal effect on TS compared to pure chickpea film (CF), with values of 6.30 MPa and 6.48 MPa, respectively. However, increasing the whey protein content led to a gradual rise in TS, peaking at 9.45 MPa in CWF4 (CP/WP ratio: 60:40). This improvement is due to enhanced protein–protein interactions and uniform dispersion within the film matrix, which strengthened the polymer network and increased cohesion [86]. These findings were further validated by the morphological uniformity observed in SEM images [82]. Conversely, excess whey protein in CWF5 reduced TS due to structural incompatibility and weaker molecular interactions—like trends reported in peanut protein–xylose films [87]. Regarding flexibility, EAB values increased with whey protein incorporation, reaching the highest value in CWF4 (157.90%), indicating enhanced chain mobility and structural compatibility [32,88]. Beyond 40% whey protein, however, flexibility declined, with CWF5 showing the lowest EAB (113.83%), likely due to structural heterogeneity and network disruption. Additionally, some studies have been conducted to compare the performance of protein-based biofilms with traditional petroleum-based packaging materials. For instance, pure poly (vinyl alcohol-co-ethylene) films containing gallic acid and umbelliferone exhibited TS and EAB values of 45 MPa and 265%, respectively [89]. Plasticized polyvinyl chloride (PVC) films, enhanced with a novel plasticizer from *Citrullus lanatus* seed oil, exhibited TS values of 17.42–18.66 MPa and EAB values of 136.18–164.32% [90]. Similarly, polypropylene-based films achieved values around 40 MPa (TS) and 300% (EAB) when processed through multiple cycles [91]. Films produced from polyethylene terephthalate (PET) via advanced molding techniques recorded a TS of 19.38 MPa [92], while additive-enriched PVC films showed a TS of 20.50 MPa and an exceptionally high EAB of 468.20% [93]. In another study, ethylene content optimization in poly (ethylene vinyl alcohol) films resulted in a TS of 107.31 MPa and an EAB of 27.12% [94]. Upon examination of the results, it can be concluded that the elongation at break value of petroleum-based films is generally higher than that of edible protein films.

### 3.7. Release of Quercetin in Different Simulants

**Figure 5:**
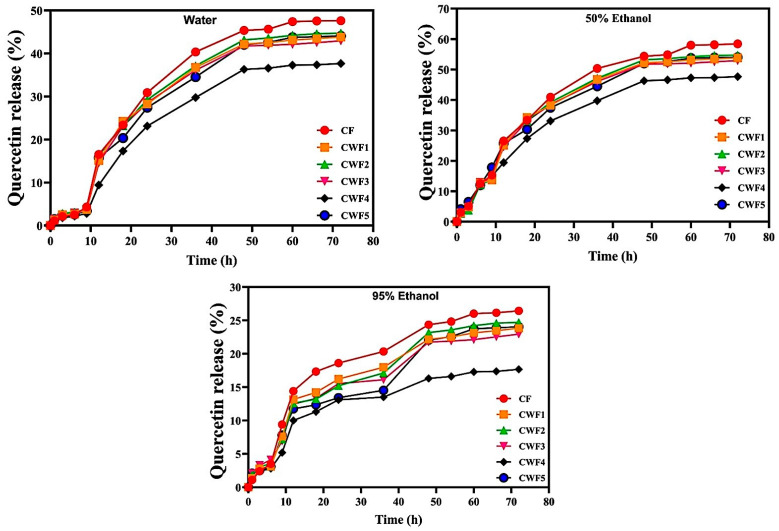
Quercetin release in food simulants (water, 50% ethanol and 95% ethanol). All results express mean ±  SD (standard deviation). CF (chickpea protein alone film), CWF1 (film containing 90% chickpea protein and 10% whey protein), CWF2 (film containing 80% chickpea protein and 20% whey protein), CWF3 (film containing 70% chickpea protein and 30% whey protein), CWF4 (film containing 60% chickpea protein and 40% whey protein), and CWF5 (film containing 50% chickpea protein and 50% whey protein).

In this study, quercetin release was monitored over a 72 h period in three different solvents: water, 50% ethanol, and 95% ethanol. The films were formulated using chickpea protein (CF) and various ratios of whey protein (CWF1–CWF5). The corresponding release profiles are presented in Figure 5. In water, quercetin release was limited during the first 6 h (2.2–3.1%) but increased to 17.3–24.2% by 18 h. Between 18 and 36 h, there was a sharp rise, reaching up to 40.34% for CF and 29.76% for CWF4. After the 36 h period, a gradual and slow release was observed in all samples, and the quercetin release rates over 72 h from these films were 47.59, 43.78, 44.77, 42.86, 37.56, and 44.34% for CF, CWF1, CWF2, CWF3, CWF4, and CWF5, respectively. This trend suggests that pure chickpea protein films (CF) are more prone to water-mediated swelling and diffusion, while the incorporation of whey protein alters the matrix structure and interaction with water. Generally, the release of active compounds from biopolymeric networks involves solvent diffusion into the film, followed by matrix swelling, and, ultimately, the release of the compound through increased free volume and enhanced diffusional pathways [95]. The observed decrease in quercetin release from CWF4 may be attributed to excessive swelling, which can lead to the formation of a denser, gel-like structure within the film. This structure may hinder diffusion by limiting the mobility of quercetin molecules, a phenomenon also described in similar systems [95]. In contrast, further increases in whey protein may have caused undesirable structural defects and facilitated release (CWF5).

In the 50% ethanol environment, all film formulations demonstrated higher release rates compared to the aqueous medium. The release profile of quercetin demonstrated a gradual and controlled pattern over time. Within the first 6 h, release exceeded 12% across all samples, indicating the onset of sustained diffusion. By 12 and 18 h, the cumulative release continued to rise steadily, reaching 19.42–26.50% and 27.30–34.26%, respectively. At 36 h, the CF film surpassed 50% release, while CWF4 remained the lowest at 39.76%. At the end of the 72 h release profile, the release rates for CF, CWF1, CWF2, CWF3, CWF4, and CWF5 were 58.43%, 53.83%, 54.71%, 52.93%, 47.66%, and 54.04%, respectively. These results suggest that the intermediate polarity of 50% ethanol promotes moderate swelling of the protein matrix and facilitates quercetin mobility [96]. These findings highlight the crucial role of matrix–solvent interactions in modulating the release behavior. Although release rates were higher than in water, CWF4 showed lower release than the other films. A more uniform, dense structure would hinder film dissolution, leading to lower release rates. Conversely, increasing the whey protein content would produce a heterogeneous film structure, making the film more susceptible to disruption due to its disordered form, which would raise the quercetin release rate. Moreover, 50% ethanol may partially disrupt protein–protein interactions or reduce the strength of the film network, allowing easier diffusion of quercetin. This dual moderate swelling and structural loosening creates favorable conditions for enhanced release [70].

Compared to the 50% ethanol and water media, 95% ethanol greatly restricted quercetin release across all film types. In the first 6 h, release stayed minimal (2.84–4.12%), and although it increased gradually over time, the final release values at 72 h were significantly lower than in other media, ranging from 17.55% for CWF4 to 26.43% for CF. These reduced release levels can be attributed to the dehydrating effect of high ethanol concentration, which increases film rigidity, suppresses hydrogen bonding, and reduces matrix permeability [96]. One of the key factors affecting the release of active compounds from films is the polarity of the solvent used. As ethanol content increases, the overall polarity of the solvent medium decreases. Consequently, the release rate remained low across all films under these conditions [97]. Among the samples, CWF4 showed the lowest release after 72 h, likely due to its more uniform and well-organized network structure, which provided a stronger barrier against diffusion. In contrast, adding additional whey protein disrupted the film structure. Although this disruption could have enhanced release, the low polarity of the ethanol-rich environment limited this effect, keeping the release rate relatively low compared to that in water and 50% ethanol medium. As previously reported, the low polarity of the solvent and the dominance of hydrophobic interactions can hinder both swelling and compound release [98].

### 3.8. Food Application

Edible packaging films provide an eco-friendly and innovative alternative for covering foods like fruits and vegetables [99]. This section investigates the potential protective effects of edible protein films on strawberries, a perishable fruit. In this context, the changes in pH, TA, WL, and the structure of both uncoated and fresh strawberries coated with various protein solutions during an 8-day storage period were investigated.

#### 3.8.1. pH and Titratable Acidity

**Figure 6:**
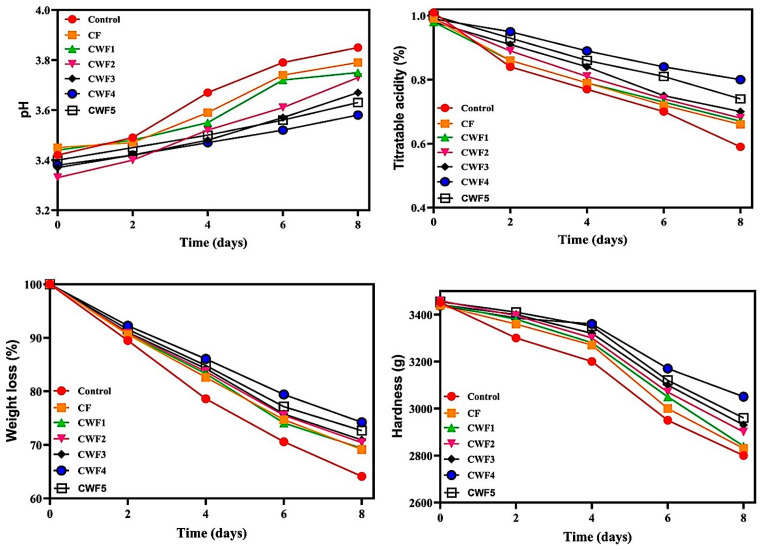
Changes in the pH, titratable acidity, weight loss, and hardness of strawberries with protein film solution coatings during storage. CF (chickpea protein alone film), CWF1 (film containing 90% chickpea protein and 10% whey protein), CWF2 (film containing 80% chickpea protein and 20% whey protein), CWF3 (film containing 70% chickpea protein and 30% whey protein), CWF4 (film containing 60% chickpea protein and 40% whey protein), and CWF5 (film containing 50% chickpea protein and 50% whey protein).

pH value is a critical factor affecting the oxidative browning of fruit tissues [100]. Changes in pH levels of uncoated and coated strawberries during storage are presented in Figure 6. The initial pH values of fresh strawberries ranged from 3.33 to 3.45, which is consistent with the typical range for different varieties (3.00–3.70) [101,102]. As shown in Figure 6, pH values increased gradually during the 8-day storage period due to the degradation of organic acids into sugars during ripening [103]. However, the rate of pH change varied among the samples. Uncoated strawberries and those coated with chickpea film (CF) exhibited the highest increase in pH. In contrast, strawberries coated with CWF4 demonstrated the greatest stability, followed by CWF5, CWF3, CWF2, and CWF1. This stability is attributed to the superior barrier properties of CWF4, which likely reduced water and gas exchange, slowing respiration and subsequent pH shifts [103]. Similar findings were reported in studies using chitosan–tea seed oil films and rice starch–curcumin nanoparticle films, both of which effectively limited pH increases during storage [104].

The alteration in acidity (TA) during storage in uncoated and coated strawberries is illustrated in Figure 6. The TA values gradually decreased during storage; however, the rate of decline was slower in coated strawberries compared to their uncoated counterparts. During 8 days of storage, TA value shifted from 1.01, 0.99, 0.98, 0.99, 0.98, 0.99 and 1.00% to 0.59, 0.66, 0.67, 0.68, 0.70, 0.80, and 0.74% for uncoated, CF-, CWF1-, CWF2-, CWF3-, CWF4- and CWF5-coated strawberry, respectively. This was due to the decrease in respiration rate and the resulting slower increase in TA because the films formed a protective O_2_ barrier on the fruit surface. Film coatings form a barrier on the outer surface of strawberries, reducing oxygen levels needed for respiration and thus decreasing metabolic activity [105]. In particular, the superior barrier properties of CWF4 helped preserve acidity by limiting gas exchange. Similar findings have been reported in studies using konjac glucomannan–pullulan films, where coated strawberries showed a slower decrease in TA compared to uncoated ones [106].

#### 3.8.2. Weight Loss and Hardness

Weight loss (WL) in strawberries primarily results from water evaporation and respiratory activity [107]. The alteration in fruit weight was monitored over 8 days (Figure 6). While all samples exhibited WL during storage, coated strawberries showed significantly lower WL than the uncoated control (*p* < 0.05). Among them, CWF4 provided the highest protection, followed by CWF5, CWF3, CWF2, and CWF1, in that order. The incorporation of whey protein into chickpea protein enhanced protein–protein interactions, leading to the formation of a denser and more cohesive network [108]. This improved matrix structure acted as an effective barrier, limiting water evaporation and gas exchange. When applied as edible coatings on strawberry fruits, the films successfully reduced moisture loss and minimized weight loss during storage. Particularly in the CWF4 formulation, the increased hydrogen bonding between proteins further strengthened the film structure, contributing to superior protective performance by slowing down moisture transfer. CWF4 film with enhanced barrier attributes helped reduce sweating, water loss, and effectively controlled weight loss in strawberries [105]. Edible films containing punicalagin were produced using apple pectin and soy protein, and their effectiveness in preserving strawberries was investigated. After seven days of storage, the uncoated sample experienced a 30% weight loss, while the film containing punicalagin showed a weight loss of 5.3% [109].

As shown in Figure 6, a decreasing trend was observed in the hardness value of all strawberries during storage. The hardness of strawberries ranged from 3438 to 3459 g at the beginning of storage and were measured between 2800 and 3050 g at the end of storage. The variation between these values on the initial and final day of storage was higher in control compared to film-coated counterparts. When the values at the end of storage were examined, the hardness of CF- and CWF1-coated strawberries was almost close to that of the control group, indicating the limited preservation abilities of these films. Among other edible films, namely CWF2, CWF3, CWF4, and CWF5, CWF4 possessed superior capabilities relative to other films. Strawberries coated with protective films retain their firmness better due to several factors: delayed ripening, reduced metabolic activity, and slower pulp degradation [44]. The protein–protein interaction within the coating help minimize water loss, inhibit oxidation, and slow down respiration, which collectively contribute to delaying microbial infection [110]. Over time, microbial activity naturally increases in strawberries, breaking down their internal structure and causing softening. Among the tested coatings, CWF4 demonstrated superior hardness retention, primarily because of its excellent barrier properties. These properties effectively reduce water loss and limit metabolic processes within the fruit, resulting in a slower decrease in firmness compared to uncoated samples. Phuong et al. (2023) reported similar results, stating that the hardness value of strawberries coated with films fabricated from chitosan and tea seed oil decreased during storage [103].

#### 3.8.3. Appearance of Strawberry During Storage

**Figure 7:**
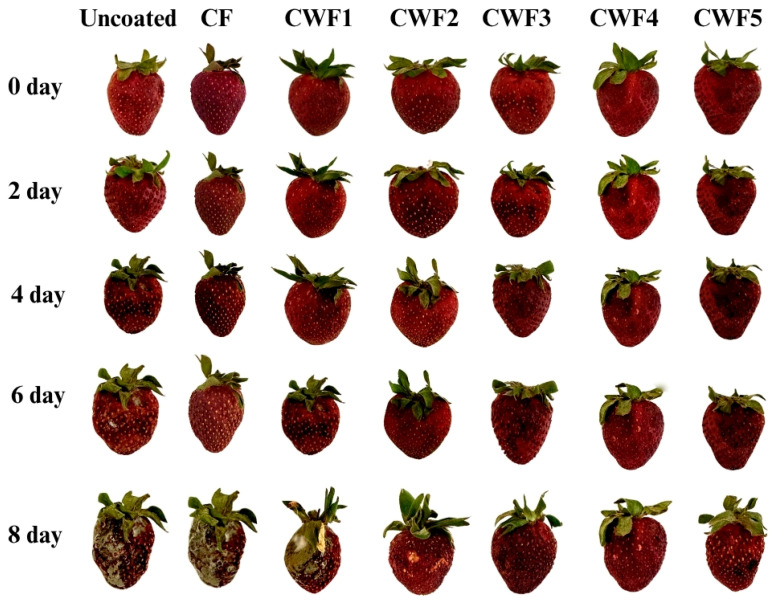
The appearance of the strawberry during storage. CF (chickpea protein alone film), CWF1 (film containing 90% chickpea protein and 10% whey protein), CWF2 (film containing 80% chickpea protein and 20% whey protein), CWF3 (film containing 70% chickpea protein and 30% whey protein), CWF4 (film containing 60% chickpea protein and 40% whey protein), and CWF5 (film containing 50% chickpea protein and 50% whey protein).

The changes in the structure of uncoated and coated strawberries during storage are given in Figure 7. It was noted that the strawberries in the control group experienced microbial spoilage starting on the fourth day of storage, and shrinkage occurred due to water loss. After this day, the strawberries were no longer edible. Moreover, in this group, quality deterioration persisted throughout storage. That said, no noticeable decrease in quality was observed in any of the film-coated samples at the end of the fourth day of storage. The films’ protective effects lasted until the end of the sixth day of storage. As for the last day of storage, CF- and CWF1- strawberries were inedible. Conversely, as the whey protein ratio increased in systems, the effect of edible films on strawberries became more evident. In other words, raising the whey protein content from 10% to 40% maximized the protective effect on strawberries. However, further increases in whey protein (50%) slightly diminished the protective effect of quercetin-containing chickpea-derived films. These results are also supported by the changes in weight loss, pH, titratable acidity, and hardness values.

## 4. Conclusions

It is evident that there has been a discernible shift towards protein (particularly plant-based) product categories within the scientific literature and sectoral applications over the past decade. Therefore, it is of considerable importance to design any product line based solely on proteins, given the current literature and industry requirements. In conjunction with these approaches, the present study involved the production of edible film systems containing exclusively protein, considering the high concentration of plant protein. This approach possesses the potential to provide innovative solutions to the scientific literature and industry by contributing to the development of sustainable and environmentally friendly food packaging materials. Incorporating whey proteins into chickpea protein-derived films containing quercetin resulted in enhancements in the properties (i.e., morphological features, water/oxygen barrier behavior, and mechanical properties) of the final products. These parameters steadily rose with the addition of whey proteins, but exceeding a specific proportion of this protein negatively impacted film quality. Peak values were obtained in CWF4. This film system also raised awareness of quercetin release in food simulators (water, 50% ethanol, and 95% ethanol) and food protection. Nonetheless, owing to their biodegradability and natural origin, the distinctive properties and mechanical strength of these films remain amenable to enhancement. Future research could enhance the mechanical and functional performance by exploring alternative cross-linking agents or novel protein combinations. Furthermore, microbial durability, biodegradability, and protective properties of quercetin-containing chickpea-derived films, particularly CWF4, can be examined in greater detail for food applications. Also, once these specific points are optimized, scale-up studies can be conducted for this film group.

## Figures and Tables

**Table 1:** The formulations of edible protein film.

Sample	Chickpea Protein (%)	Whey Protein (%)	Quercetin (mg/g Dry Matter)	Glycerol (g/g Protein)
CF	100	0	50	0.30
CWF1	90	10	50	0.30
CWF2	80	20	50	0.30
CWF3	70	30	50	0.30
CWF4	60	40	50	0.30
CWF5	50	50	50	0.30

**Table 2:** Amino acids composition of chickpea and whey proteins.

	Samples	
Essential Amino Acids	Chickpea Protein (%)	Whey Protein (%)
Histidine	1.26 ± 0.00	0.41 ± 0.00
Leucine	1.73 ± 0.05	1.16 ± 0.01
Isoleucine	0.01 ± 0.00	0.85 ± 0.00
Lysine	4.23 ± 0.07	1.41 ± 0.01
Methionine	1.30 ± 0.02	1.17 ± 0.04
Phenylalanine	2.28 ± 0.05	0.73 ± 0.03
Threonine	1.57 ± 0.05	0.56 ± 0.00
Tryptophan	2.65 ± 0.03	0.06 ± 0.00
Valine	1.44 ± 0.06	1.73 ± 0.03
Sum	16.48 ± 0.10	8.09 ± 0.08
Non-essential amino acids	Chickpea protein (%)	Whey protein (%)
Alanine	2.20 ± 0.05	60.02 ± 0.25
Anserine	2.74 ± 0.00	7.78 ± 0.05
Arginine	34.16 ± 0.24	0.78 ± 0.03
Argininosuccinic acid	0.89 ± 0.01	0.00 ± 0.00
Asparagine	3.28 ± 0.02	0.59 ± 0.00
Aspartic acid	2.32 ± 0.07	1.05 ± 0.02
Beta-alanine	0.17 ± 0.00	0.07 ± 0.00
Beta amino iso butyric acid	0.20 ± 0.05	0.11 ± 0.02
Citrulline	2.11 ± 0.05	0.18 ± 0.01
Etanolamine	4.24 ± 0.10	0.65 ± 0.00
Gamma amino butyric acid	0.15 ± 0.02	0.11 ± 0.02
Glutamic acid	12.30 ± 0.11	1.82 ± 0.05
Glutamine	1.08 ± 0.01	1.65 ± 0.01
Glycine	4.65 ± 0.09	4.81 ± 0.06
Histamine	0.01 ± 0.00	0.50 ± 0.05
Hydroxyproline	0.70 ± 0.04	0.45 ± 0.01
Ornithine	4.24 ± 0.09	0.43 ± 0.02
Proline	3.67 ± 0.10	3.71 ± 0.04
Sarcosine	0.41 ± 0.02	4.69 ± 0.04
Serine	2.21 ± 0.04	1.77 ± 0.03
Thia proline	0.61 ± 0.03	0.03 ± 0.00
Tyrosine	1.18 ± 0.06	0.41 ± 0.02
Sum	83.52 ± 0.25	91.91 ± 0.19

Results are given as mean ± standard deviation (*n* = 3).

**Table 3:** Physicochemical features of edible films.

Sample	Moisture Content (%)	Thickness (mm)	L*	a*	b*	Opacity (Abs/mm)
CF	17.86 ± 0.11 ^b^	0.10 ± 0.01 ^c^	82.08 ± 0.01 ^f^	−0.28 ± 0.01 ^a^	28.37 ± 0.01 ^a^	1.73 ± 0.01 ^d^
CWF1	17.99 ± 0.04 ^b^	0.10 ± 0.01 ^c^	84.15 ± 0.01 ^e^	−0.34 ± 0.02 ^a^	27.00 ± 0.18 ^b^	1.85 ± 0.01 ^bc^
CWF2	17.76 ± 0.31 ^b^	0.11 ± 0.01 ^bc^	84.73 ± 0.05 ^d^	−0.44 ± 0.02 ^b^	26.07 ± 0.08 ^c^	1.76 ± 0.01 ^d^
CWF3	18.01 ± 0.47 ^b^	0.12 ± 0.01 ^b^	85.51 ± 0.02 ^c^	−0.66 ± 0.01 ^c^	25.54 ± 0.05 ^d^	1.84 ± 0.01 ^c^
CWF4	16.12 ± 0.32 ^c^	0.10 ± 0.01 ^c^	85.94 ± 0.01 ^b^	−0.70 ± 0.05 ^cd^	24.89 ± 0.03 ^e^	1.93 ± 0.01 ^b^
CWF5	20.18 ± 0.23 ^a^	0.15 ± 0.01 ^a^	86.32 ± 0.21 ^a^	−0.76 ± 0.04 ^d^	24.35 ± 0.08 ^f^	2.55 ± 0.01 ^a^

All results express mean ±  SD (standard deviation). ^a–f^ Lowercase letters in the same column present statistical differences (*p* < 0.05). CF (chickpea protein alone film), CWF1 (film containing 90% chickpea protein and 10% whey protein), CWF2 (film containing 80% chickpea protein and 20% whey protein), CWF3 (film containing 70% chickpea protein and 30% whey protein), CWF4 (film containing 60% chickpea protein and 40% whey protein), and CWF5 (film containing 50% chickpea protein and 50% whey protein).

**Table 4:** Effect of different solvents on the swelling ratio and solubility of films.

Sample	Solubility (%)			Swelling Ratio (%)		
	Water	50% Ethanol	%95 Ethanol	Water	50% Ethanol	%95 Ethanol
CF	73.05 ± 0.88 ^a^	69.97 ± 0.66 ^a^	35.24 ± 0.16 ^a^	171.85 ± 2.13 ^d^	159.95 ± 0.86 ^d^	52.41 ± 0.37 ^f^
CWF1	70.33 ± 0.68 ^bc^	67.67 ± 0.31 ^b^	33.01 ± 0.31 ^c^	186.09 ± 2.02 ^c^	171.12 ± 0.94 ^c^	58.12 ± 0.32 ^d^
CWF2	68.23 ± 0.31 ^d^	67.00 ± 0.33 ^bc^	32.06 ± 0.07 ^d^	188.79 ± 2.20 ^c^	172.12 ± 0.47 ^c^	65.05 ± 0.25 ^c^
CWF3	69.06 ± 0.62 ^cd^	66.29 ± 0.23 ^c^	31.20 ± 0.36 ^e^	266.32 ± 3.02 ^b^	246.00 ± 1.20 ^b^	70.16 ± 0.41 ^b^
CWF4	60.62 ± 0.74 ^e^	58.40 ± 0.27 ^d^	27.91 ± 0.52 ^f^	282.02 ± 1.83 ^a^	260.06 ± 0.87 ^a^	66.06 ± 0.40 ^a^
CWF5	71.15 ± 0.50 ^b^	69.39 ± 0.50 ^a^	33.95 ± 0.23 ^b^	164.16 ± 1.67 ^e^	153.05 ± 0.72 ^e^	54.45 ± 0.31 ^e^

All results express mean ±  SD (standard deviation). ^a–f^ Lowercase letters in the same column present statistical differences (*p* < 0.05). CF (chickpea protein alone film), CWF1 (film containing 90% chickpea protein and 10% whey protein), CWF2 (film containing 80% chickpea protein and 20% whey protein), CWF3 (film containing 70% chickpea protein and 30% whey protein), CWF4 (film containing 60% chickpea protein and 40% whey protein), and CWF5 (film containing 50% chickpea protein and 50% whey protein).

## Data Availability

The datasets from the current study are available from the corresponding author upon reasonable request.
